# Combining measures of immune infiltration shows additive effect on survival prediction in high-grade serous ovarian carcinoma

**DOI:** 10.1038/s41416-020-0822-x

**Published:** 2020-04-06

**Authors:** Anne Montfort, Rowan J. Barker-Clarke, Anna M. Piskorz, Anna Supernat, Luiza Moore, Sarwah Al-Khalidi, Steffen Böhm, Paul Pharoah, Jacqueline McDermott, Frances R. Balkwill, James D. Brenton

**Affiliations:** 1 Barts Cancer Institute, Queen Mary University of London, London, UK; 2 Cancer Research UK Cambridge Institute, Cambridge, UK; 3 Department of Oncology, University of Cambridge, Cambridge, UK; 4 Laboratory of Translational Oncology, Intercollegiate Faculty of Biotechnology, Medical University of Gdańsk, 80-211 Gdańsk, Poland; 5 Department of Public Health and Primary Care, Centre for Cancer Genetic Epidemiology, University of Cambridge, Cambridge, UK; 6 Department of Pathology, University College London Hospital, London, UK; 7 Cambridge University Hospitals NHS Foundation Trust, Cambridge, UK

**Keywords:** Ovarian cancer, Imaging the immune system

## Abstract

**Background:**

In colorectal and breast cancer, the density and localisation of immune infiltrates provides strong prognostic information. We asked whether similar automated quantitation and combined analysis of immune infiltrates could refine prognostic information in high-grade serous ovarian carcinoma (HGSOC) and tested associations between patterns of immune response and genomic driver alterations.

**Methods:**

Epithelium and stroma were semi-automatically segmented and the infiltration of CD45RO^+^, CD8^+^ and CD68^+^ cells was automatically quantified from images of 332 HGSOC patient tissue microarray cores.

**Results:**

Epithelial CD8 [*p* = 0.027, hazard ratio (HR) = 0.83], stromal CD68 (*p* = 3 × 10^−4^, HR = 0.44) and stromal CD45RO (*p* = 7 × 10^−4^, HR = 0.76) were positively associated with survival and remained so when averaged across the tumour and stromal compartments. Using principal component analysis, we identified optimised multiparameter survival models combining information from all immune markers (*p* = 0.016, HR = 0.88). There was no significant association between PTEN expression, type of *TP53* mutation or presence of *BRCA1/BRCA2* mutations and immune infiltrate densities or principal components.

**Conclusions:**

Combining measures of immune infiltration provided improved survival modelling and evidence for the multiple effects of different immune factors on survival. The presence of stromal CD68^+^ and CD45RO^+^ populations was associated with survival, underscoring the benefits evaluating stromal immune populations may bring for prognostic immunoscores in HGSOC.

## Introduction

There is a heterogeneous immune response in the tumour microenvironment of HGSOC, but the presence of intra-epithelial CD8^+^ T cells is consistently associated with improved survival.^1,2^ Prolonged survival is also associated with the presence of B cells and other immune cells, including CD45RO^+^ memory cells.^3–6^

Prognostic scoring has predominantly focused on the presence, absence or semi-quantitative analysis of immune cells in tumour epithelium.^7^ Moreover, cell densities of lymphocytes, macrophages and dendritic cells have also been shown to be prognostic in breast, ovarian and lung cancer without restricting analyses to malignant epithelial cell areas.^8–10^ Approaches that combine analyses of multiple immune infiltrates, such as the CD3/CD8 immunoscore in colorectal cancer,^11^ have not yet been developed for ovarian cancer prognosis.

The notion that anti-tumoural immune responses may be altered by tumour cell-intrinsic factors is supported by several observations. Detailed temporal and spatial histological and genomic studies in a single patient with high-grade serous ovarian carcinoma (HGSOC) showed that Wnt signalling was upregulated in a progressing tumour nodule, a phenomenon correlated with focal immunosuppression.^12^ Mutations in the *TP53* and *BRCA1*/*BRCA2* genes as well as loss of *PTEN* expression are driver events in HGSOC development.^13^ HGSOC cases with *BRCA1* mutations have increased CD8 and CD20 intra-epithelial infiltrates,^14,15^ suggesting that loss of homologous recombination and DNA damage may prime immune responses. Whether different classes of *TP53* mutations alter immune infiltration in HGSOC remains unknown, but non-synonymous mutations in *TP53* may have gain-of-function or other cellular effects distinct from loss-of-function mutations.^16^ Mutant p53 protein may drive B cell responses and auto-antibody production.^17^ Loss of *PTEN* expression in melanoma was associated with both reduced T cell infiltration and resistance to immune checkpoint inhibitors,^18^ but these associations have not been investigated in HGSOC.

Despite the strong association of CD8^+^ infiltrate with prognosis, routine immunoscoring for ovarian cancer is not performed in the clinic and development of automatic cellular recognition tools could be beneficial for high-throughput pathology workflows. To test the hypothesis that a more integrated analysis of cytotoxic, mature and organised immune responses in the tumour microenvironment might have greater prognostic value in HGSOC, we developed new image analysis methods and workflows to test the single and integrated analysis of CD8^+^, CD45RO^+^ and CD68^+^ in the malignant epithelium and adjacent stroma. The secondary aim of this study was to test for association between quantitative measures of CD8^+^, CD45RO^+^ and CD68^+^ cells and driver genomic alterations in *BRCA1*, *BRCA2*, *PTEN* and different classes of *TP53* mutation.

## Materials and methods

### Patients

Samples from 570 patients from the prospective SEARCH ovarian cancer population-based study were used to construct tissue microarrays (TMAs).^19^ Ethical approval was granted by the Eastern Multicenter Research Ethics Committee. Among the samples from 570 patients with primary epithelial ovarian tumours, 332 were high-grade serous ovarian cancer patients. All cases underwent detailed histopathological review by a gynaecological pathologist (J.Mc.D.). Patients were staged as having localised, regional or distant disease (L/R/D).^20^

### Immunohistochemistry

Microarray slides composed of formalin-fixed paraffin-embedded ovarian tumour cores were dewaxed and rehydrated prior to heat-induced epitope retrieval using a pressure cooker and a citrate-based antigen unmasking solution (Vector Laboratory). Detection of CD8^+^ T cells, CD45RO^+^ memory lymphocytes and CD68^+^ macrophages was performed using the mouse anti-human CD8 (clone C8/144B, Dako), mouse anti-human CD45RO (clone UCLH, Dako) and mouse anti-human CD68 (clone M0876, Dako) antibodies, using ultrasensitive Polymer-HRP IHC Detection system (Biogenex). Immunohistochemical protocols and slide hybridisations were carried out manually. Sections were counterstained with haematoxylin and mounted with DPX mounting medium (Sigma). Previously published PTEN immunostaining data was used where high PTEN expression was considered to be positive staining and low expression to be weak, heterogeneous or negative staining, respectively.^21^

### Mutation analysis

The coding regions of *TP53* were sequenced by tagged-amplicon next-generation sequencing as previously described^22^ and confirmed by immunohistochemical analysis using a 4-tier core system.^23^ Sequencing of germline mutations in the *BRCA1* and *BRCA2* genes was performed as previously described.^24^

### Immune cell quantification

Stained slides were scanned using the Panoramic Slash Scanner (3D Histech). The number of CD8^+^ and CD45RO^+^ cells per mm^2^ of epithelial and stroma areas, as well as the percentage of epithelial and stromal areas covered by CD68 staining, were digitally determined using the Tissue Studio software (Definiens™). Definiens image analysis algorithms for detection of epithelial and stromal areas were trained and the segmentation for each core was manually refined by two researchers, including a consultant gynaecological-histopathologist (J.Mc.D.). Supplementary Fig. 1 shows examples of classifications of tissue regions and cell detection and the entire data set, including these epithelial and stromal assignments, can be downloaded from the repository.

### Statistical analyses

R (version 3.5.1) was used for statistical analysis and an R markdown document containing the entire data set allowing for performing all analyses is available (https://bitbucket.org/jamesdbrenton/search-montfort/src/master/). Quality checking for spatial bias across TMAs and effects of varying tissue area was carried out upon all cores and across TMAs using heatmaps and Shapiro–Wilk tests. All count data were transformed to log base 10 after adding a small offset to zero values. Wilcoxon’s signed-rank test was used to compare the mean infiltrate between groups. Continuous data were presented as median and interquartile range (IQR) and groups were compared by the Kruskal–Wallis and pairwise Kruskal–Wallis tests. Discrete data were presented as count and percentage.

Cox proportional hazard regression analysis was applied to assess the effect of each infiltrate on overall survival. The functional form of each of the immune variables was assessed using comparison with cubic splines. The best approximations to the functional forms were carried forward for the Cox models. The clinical variables of age at diagnosis, menopause status and stage were available for the cohort and were included in the analysis. Univariable Cox regressions were used to identify best-fitting variables for the final multivariable Cox regression model. The refined model was compared with a combined multivariable Cox regression model including all immune infiltrates. Hazard ratios (HRs) refer to a single unit increase in continuous variables. The proportional hazards assumption was tested and satisfied in all cases using Schoenfeld residuals. The Kaplan–Meier analysis (with log-rank test) was applied to illustrate survival differences graphically. Two-sided *p* values <0.05 were used to indicate statistical significance. Principal component analysis (PCA) using the R package prcomp was used to extract the independent components of variance between patients. The package prcomp uses singular value decomposition and the variables were scaled to have unit variance before creating composite linear independent variables. These were then passed forward to the survival modelling. The Akaike information criterion (AIC) was used to compare the performance of survival models, which includes a penalty on the number of terms to reduce overfitting. Bonferroni *p* value corrections were carried out for all multiple testing. *P* < 0.05 was considered significant for all analyses.

## Results

### Patient characteristics

Supplementary Fig. 2 shows the REMARK diagram for this study and Supplementary Table 1 shows the clinical characteristics of the 332 HGSOC patients from the study cohort. Immunohistochemical analyses on TMAs were performed to detect CD8^+^, CD45RO^+^ and CD68^+^ cells in tissue cores from primary ovarian specimens. One hundred and fifty-two HGSOC cases were available for analysis after quality assurance, data cleaning and the reduction of the data set to only cases with complete results for CD8, CD45RO and CD68 staining in both epithelium and stroma, as well as survival data.

Tagged-amplicon sequencing was performed on 248 cases and *TP53* mutation was detected in 231 samples (93%) (Supplementary Table 1). Previously published data for germline *BRCA1* and *BRCA2* mutation and PTEN expression were available for 297 and 155 cases, respectively.^18,22^

### Digital pathology analysis of tumour composition and immune cell densities

**Fig. 1:**
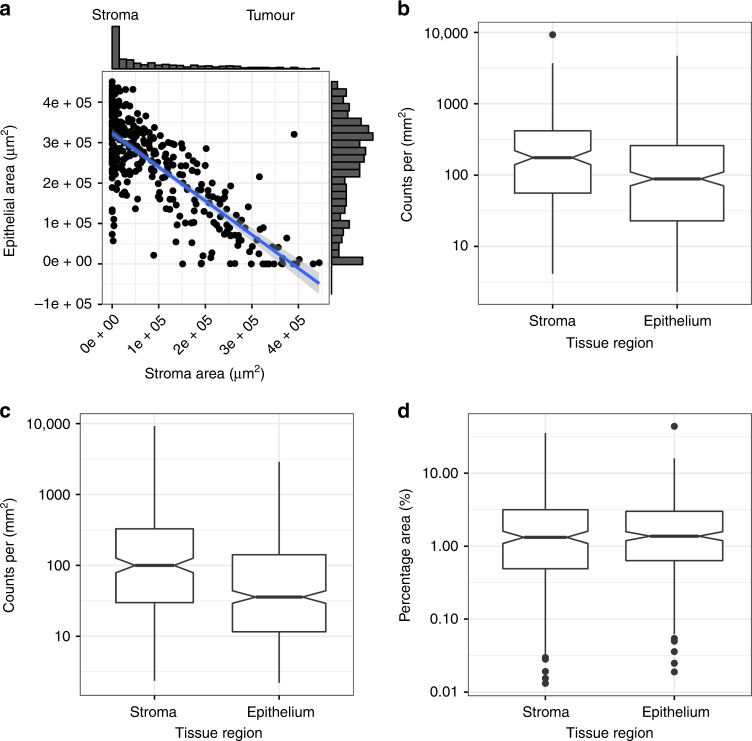
Proportions of immune cells vary between epithelial and stroma areas of tumours. **a** Scatter plot of the average stroma and epithelial tumour areas for each patient. **b**–**d** show respectively the distribution of densities of CD8^+^, CD45RO^+^ and CD68^+^ cell in epithelium and stroma. CD8^+^ and CD45RO^+^ densities were defined as counts per mm^2^ and CD68^+^ as the percentage of tissue stained for this marker. Notches on box plots extend 1.58 × IQR/sqrt(*n*) and approximate the 95% confidence interval for the median. Box plot whiskers extend to 1.5 × IQR.

Image analysis software was used to determine the area of tumour epithelium and stroma in each core (Fig. 1 and Supplementary Fig 1). Of 964 images representing 332 HGSOC patients, 69 patients (20.8%) had images that contained malignant epithelium but no stroma; 250 patients (75.3%) had images that contained epithelium and >1% adjacent stroma and 13 patients (3.9%) had images containing no tumour epithelium (Fig. 1a). The median proportion of epithelium and stroma was 85.1% (IQR 51–100%) and 14.9% (IQR 0–49%), respectively. We expected the proportion of tumour in a sample to be correlated with p53 mutant allele fraction, a measure of sample purity, and found them to be positively correlated (*R*^2^ = 0.25, *p* = 0.0004).

Quantitative densities of all immune populations were then generated automatically through image analysis, the quantitative assessment of CD8^+^ T cell, CD45RO^+^ memory lymphocyte and CD68^+^ macrophage densities in each compartment are shown in Supplementary Fig. 1. The relationship between the fraction of tumour in a core and the density of immune infiltration in the epithelium was examined. Intra-epithelial CD8^+^ and CD45RO^+^ densities were weakly correlated with the purity/tumour fraction of the sample (*R*^2^ = 0.17, *p* = 0.003 and *R*^2^ = 0.16, *p* = 0.006), but CD68^+^ epithelial density was not.

The three immune populations in our samples showed moderate to strong correlation between epithelium and stroma (Supplementary Fig. 3 and Supplementary Table 2). Samples with low density of stromal immune populations generally had low density of epithelial infiltrate and vice versa. The distribution of densities of immune populations within tumour epithelium and stromal areas were compared (Fig. 1b–d). The density of CD8^+^ and CD45RO^+^ cells were significantly higher in stroma than in tumour epithelium (*p* = 0.005 and *p* = 0.004, respectively; Welch’s *t* test), but not significantly different for CD68^+^ cells.

In describing the patterns of immune infiltrate, the terms immune inflamed, immune desert and immune excluded have been used to describe varying T cell infiltration based on histological and transcriptional analyses.^12,25–27^ Immune-inflamed and immune-desert patterns reflect high positive or negative correlations between all infiltrates, but T cell exclusion describes tumours where CD8^+^ cells are significantly absent from tumour epithelium while still being present in the surrounding stroma.^28,29^ Given the higher infiltration in stroma than epithelial compartments, we defined immune cell exclusion as a 10-fold difference between tumour epithelium and stromal infiltration as the standard deviation of the log 10-transformed counts was ~1. CD8^+^ exclusion was present in 20 (10.6%) cases and 36 (20%) cases had CD45RO^+^ exclusion. No cases had significant exclusion of CD68^+^ infiltrate from tumour epithelium. Notably none of the cases had both CD8^+^ and CD45RO ^+^ exclusion.

### Stromal CD68^+^ and CD45RO^+^ densities are the strongest individual prognostic markers

Survival was modelled using Cox proportional hazards and the relationship between the immune variables and survival was found to be approximately log linear. The clinical variables accompanying the cohort were age at diagnosis, stage and menopause status and the relationship between age and survival was found to be approximately linear (see Methods, Supplementary Fig. 4 and Supplementary Table 3).

**Fig. 2:**
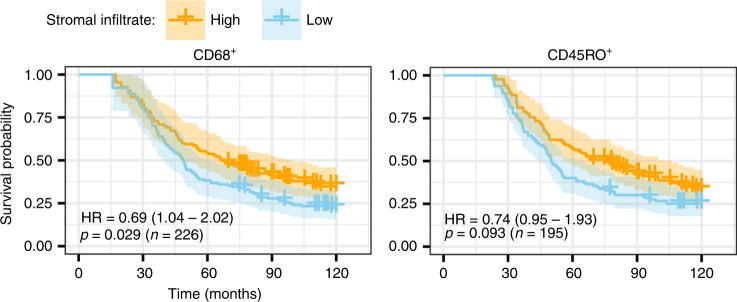
Survival analysis of HGSOC patients relative to the density of CD68^+^ macrophages and CD45RO^+^ cells in their stroma. Kaplan–Meier survival curves using cut point of median density of stromal CD68^+^ macrophages and CD45RO^+^ memory T cells using left truncation and right censoring. Median entry to the study for all patients after diagnosis was 26.4 months. Median follow-up time from diagnosis to exit or death was 105.1 months.

Univariable analysis showed improved survival with increasing stromal density of CD45RO^+^ [HR 0.76, 95% confidence interval (CI): 0.65–0.90, *p* = 0.001] and CD68^+^ (HR 0.53, 95% CI: 0.34–0.81, *p* = 0.003) (Table 1). Modelling each immune variable with stage showed improved predictive value for epithelial CD8^+^ density (HR = 0.83, *p* value=0.027) as well as stromal CD68^+^ and CD45RO^+^ density and epithelial CD45RO^+^ density (Table 1). Figure 2 and Supplementary Fig. 5 shows illustrative Kaplan–Meier survival curves for high and low stromal and epithelial CD68^+^, CD45RO^+^ and CD8^+^ densities.

**Table 1:** Hazard ratios from the Cox proportional model for all infiltrates in all regions and averaged across the whole core in HGSOC, measured as log 10 (counts per mm^2^).

Functional form	Evaluable cases	Tissue compartment	Univariable		Multivariable^a^ (adjusted for stage)	
			HR	*p* Value	HR	*p* Value
CD8^+^						
Log 10	301	Epithelium	0.89	0.15	0.83	0.027
Log 10	202	Stroma	0.97	0.74	0.93	0.40
Log 10	315	Average	0.79	0.010	0.72	0.0006
CD45RO^+^						
Log 10	290	Epithelium	0.86	0.033	0.85	0.022
Log 10	196	Stroma	0.76	0.001	0.76	0.0007
Log 10	306	Average	0.82	0.006	0.80	0.003
CD68^+^						
Linear	293	Epithelium	0.99	0.67	0.99	0.43
Log 10	226	Stroma	0.53	0.003	0.44	0.0003
Log 10	308	Average	0.67	0.042	0.62	0.017
Stage						
–	312	Localised	1	0	1	0
		Regional	1.47	0.26	1.15	0.25
		Distant	3.96	≪0.001	5.58	≪0.001
		Unstaged	3.35	<0.001	3.34	≪0.001

^a^Multivariable analysis was adjusted for stage.

In cores with <1% stroma, epithelial CD8^+^ malignant epithelial infiltrate remained an independent prognostic factor, but epithelial CD45RO^+^ density was not significant (Supplementary Table 4).

In clinical reporting, quantifying immune populations in exclusively tumour epithelium is technically challenging and time consuming. We tested the effects of using the average density of each marker averaged across both tumour and stromal regions from each core (Table 1). Averaging the tissue density of CD8^+^ increased the strength of the associated HR and significance of the model (HR = 0.79, *p* value = 0.010) indicating increased prognostic value over quantitation of individual epithelial and stromal infiltrates. Supplementary Fig. 5 shows illustrative Kaplan–Meier survival curves for high and low CD68^+^, CD45RO^+^ and CD8^+^ densities over combined epithelium and stroma compartments.

Multivariable Cox regression analysis including all infiltrates and stage was carried out on patients with complete data for all infiltrates (*n* = 152) (Table 2). In this model, only stage and CD68^+^ stromal infiltrate were significant predictors of survival.

**Table 2:** Multivariable Cox regression hazard ratios and associated *p* values for a model with all infiltrates and stage and for a reduced model with most significant variables only (*n* = 152).

	Multivariable (all combined)		Refined model	
	HR	*p* Value	HR	*p* Value
CD8^+^				
Epithelium	0.96	0.81	–	–
Stroma	1.07	0.63	–	–
CD45RO^+^				
Epithelium	1.12	0.37	–	–
Stroma	0.83	0.09	0.68	0.11
CD68^+^				
Epithelium	1.16	0.63	–	–
Stroma	0.53	0.038	0.88	0.17
Stage				
Localised	1	0	1	0
Regional	2.00	0.16	2.03	0.14
Distant	4.82	≪0.001	4.70	0.0001
Unstaged	8.15	≪0.001	8.25	0.0001

We then refined the model by removing the least significant elements (defined as those with *p* > 0.1) (Table 2, *n* = 152). Interestingly, we found that the *p* values for CD68^+^ and CD45RO^+^ stromal infiltrates in the refined model become less significant and the hazard ratios are attenuated in comparison to both the univariable regression and the full model. This is likely due to the inability of Cox regression to distinguish with confidence whether stromal CD45RO^+^ or CD68^+^ density is the most significant predictor when there is a strong correlation between all the immune variables.

### Principal components of the combined immunospace describe biologically interpretable effects

As the three types of immune infiltrate vary continuously across epithelium and stroma these variables can be regarded as six dimensions of an ‘immunospace’ (three infiltrates, two localisations). Given the strong correlations between infiltrates (Supplementary Fig. 3), these immune variables are not independent. We used PCA to determine the independent patterns across these dimensions, using the 152 patients for whom complete data were available. PCA transformed the six correlated infiltrate variables into six independent axes with the first component containing the largest proportion of variance (60%) in the data set (Supplementary Table 5).

In principal component 1 (PC1), the weightings of all immune infiltrates are positive and similar in magnitude (Supplementary Table 5). This indicates that as one infiltrate increases so do all the others and represents the degree of coordinated immune response. The remaining PCs characterise additional patterns across immune infiltrates independent of PC1. The additive contribution of PC2 characterises negative correlation between CD8^+^ infiltrates and CD68^+^ macrophages and CD45RO^+^ memory cells. PC3 characterises additional variation where epithelial and stromal infiltrates are negatively correlated, the most positive values of PC3 correspond to high infiltration in tumour epithelium compared to stroma and the most negative values of PC3 correspond to the opposite, the aforementioned immune exclusion.

**Fig. 3:**
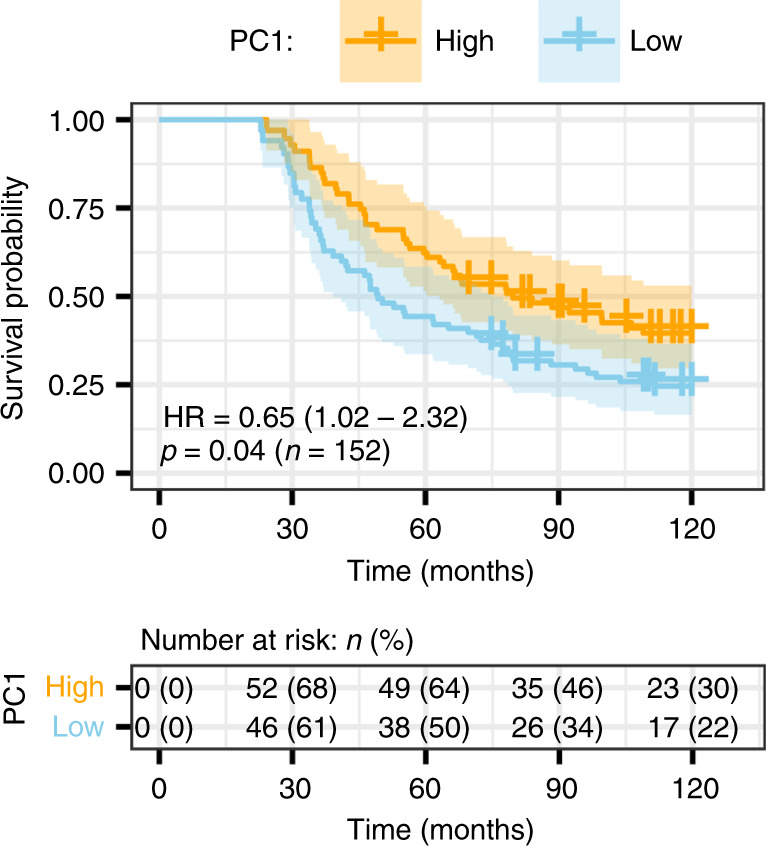
Survival analysis by principal component 1 (PC1). Kaplan–Meier analysis using median cut point for PC1 with left truncation for study entry variables and right censored at 120 months. Median entry to the study for all patients after diagnosis was 26.4 months. Median follow-up time from diagnosis to exit or death was 105.1 months.

Supplementary Fig. 6 shows representative images with the largest magnitudes of PC1, PC2 and PC3 to visually illustrate the patterns described above. The variance in the remaining PCs (4–6) is smaller and less informative. Variance in PC4 is predominantly from CD45RO^+^ stromal density, PC5 is from CD45RO^+^ epithelial density and PC6 is from CD68^+^ epithelial density. Patients are plotted by their PC1 and PC2 values in Supplementary Fig. 7. Cox proportional hazard regression was used to assess whether these PCs were predictive of survival. Only PC1 was an independent predictor of survival in our cohort and was associated with improved survival (univariate: HR = 0.89, *p* value = 0.024; PC1 + stage: HR: 0.88, *p* value = 0.019) (Supplementary Table 6), reflecting the good prognosis of a strong coordinated immune response. The association of this PC with survival is also illustrated graphically using Kaplan–Meier curves in Fig. 3.

Cox regression survival models were also calculated on all combinations of PCs and stage. The AIC was used to compare the performance of these survival models and includes a penalty on the number of terms to reduce overfitting (Supplementary Fig. 8). The model combining stage, PC1 and PC5, had the best performance for predicting overall survival. The improvement with the addition of PC5 shows that the addition of this PC has a suppressor effect in the model, increasing the significance of other variables when included. This demonstrates that survival is predominantly determined by the coordinated immune response and further variation in survival from this trend can be encoded by the quantity of epithelial CD45RO^+^ infiltrate. Interestingly, the models that contained stage and either stromal CD45RO^+^ or CD68^+^ infiltrate contained a similar amount of information about patient survival as the one that contained stage and PCs 1 and 5. In our cohort, the density of CD68^+^ and CD45RO^+^ stromal infiltrates are therefore the best single infiltrates for survival modelling.

### Are genetic defects associated with HGSOC driving individual infiltrates or the coordinated immune response in tumours?

The presence of germline *BRCA2* mutations was significantly associated with lower CD8^+^ cell density than patients with a *BRCA1* after multiple testing correction (Table 3). There was no significant association between the quantity of CD45RO^+^ or CD68^+^ infiltrate and the mutational status of either *BRCA1* or *BRCA2* genes (Table 3). No significant association was detected between *TP53* gain-of-function (GOF) and loss-of-function (LOF) mutations or PTEN expression and the densities of CD8^+^, CD45RO^+^ and CD68^+^ cells in the epithelium or stroma (Table 3). Similarly, changes in the PCs were not significantly associated with PTEN expression, *TP53* GOF or LOF mutation or germline *BRCA1*/*BRCA2* mutation status (Table 3).

**Table 3:** Association between immune infiltrate and genetic alterations in HGSOC tumours.

	*BRCA1/BRCA2*	p53 GOF/LOF	PTEN
CD8^+^			
Epithelium	0.04	0.54	0.67
Stroma	0.17	0.29	0.07
Average	0.17	0.53	0.84
CD45RO^+^			
Epithelium	0.20	0.42	0.92
Stroma	0.46	0.84	0.82
Average	0.10	0.36	0.87
CD68^+^			
Epithelium	0.43	0.72	0.79
Stroma	0.78	0.85	0.65
Average	0.68	0.66	0.41
Principal component			
1	0.81	0.80	0.55
2	0.10	0.26	0.17
3	0.27	0.58	0.51
4	0.58	0.99	0.17
5	0.76	0.88	0.79
6	0.97	0.44	0.18

*P* values were associated with Kruskal–Wallis test for detecting differences in mean ranks of immune infiltrate in patients grouped by mutation in *BRCA1*, *BRCA2* or not-detected, p53 GOF or LOF and PTEN high or low in HGSOC.

## Discussion

Infiltration of immune cells in HGSOC tumours has previously been correlated to prognosis for patients.^1,30^ However, translation of these tools to the clinic has been impeded by the lack of standardised and reliable quantitation methods. In this work, we used a semi-automated approach to quantify the CD8^+^, CD45RO^+^ and CD68^+^ infiltrates in both stromal and epithelial areas of primary HGSOC tumours. We found that immune infiltration in the tumour microenvironment was continuously distributed across a wide range and CD8^+^, CD45RO^+^ and CD68^+^ infiltrates were strongly correlated. T cell exclusion from epithelial regions has been previously reported.^29^ We only observed epithelial exclusion of CD8^+^ or CD45RO^+^ in 10% and 20% of patients, respectively. These groups were mutually exclusive, suggesting that immune exclusion from epithelial or stromal regions, is a tumour-specific effect that is distinct from coordinated multi-infiltrate immune responses.

Our results for the positive prognostic effects of epithelial CD8^+^ T cells are consistent with the large study by Goode et al.,^2^ who described a near log-linear relationship between the density of epithelial CD8^+^ T cells and survival. However, our observations also show that averaging CD8 density across the total core (epithelial and stromal areas) improves survival prediction. The alternative possibility that epithelial and stroma compartments have been mis-assigned is highly unlikely as these assignments were all reviewed by a gynaecological pathologist. The combination of both stroma and tumour epithelium potentially provides a better representation of the dynamics of epithelial infiltration by including cells that may be poised to infiltrate.

CD45RO is a memory marker for T and B cells. In the tumour microenvironment, we and others^5,31,32^ have shown that CD4^+^ and CD8^+^ T cells, myeloid cells and B cells are mainly located in the stroma of tumours, whereas CD8^+^ T cells can be found in both areas. Meta-analyses investigating the prevalence of CD45RO in solid cancers reported that high CD45RO infiltrate was associated with better prognosis for patients.^33^ We confirmed these results in HGSOC and demonstrated that the quantity of stromal and average densities of CD45RO^+^ cells over the whole core are prognostic. We also demonstrated that epithelial CD45RO^+^ density was a significant predictor of survival in cores with >1% stroma, but not in those with <1% stroma. This result emphasises the need to contextualise epithelial infiltrate and the confounding nature of tumour composition on survival impact.

The link between tumour infiltration with macrophages and patient survival is more complex. Previous studies demonstrated the positive predictive value of classically activated (M1) over tumour-promoting (M2) macrophages in ovarian cancer.^9,34^ However, no significant association was found between the level of intra-epithelial CD68 infiltrate and patient survival.^4^ Our study confirms this latter result and also demonstrates a significant association between a high coverage of stromal areas with CD68^+^ macrophages and improved overall survival. This result is in line with a recent study showing a high infiltration of CD68^+^ macrophages at the invasive front of tumour sections from colorectal cancer patients is correlated to better response to chemotherapy.^35^ Whereas tumour-associated macrophages (TAM) promote cancer progression via stimulation of multiple processes including angiogenesis, inflammation and immune escape, it now appears evident that the phenotype of TAM changes in different areas of the tumour.^36,37^ Taking into account the seemingly contradictory results obtained on this subject, additional studies aiming at deciphering the precise role distinct subpopulations of macrophages infiltrating different areas of tumours play in cancer progression is required.

All three infiltrates were also found to be predictive in multivariate analyses adjusted for tumour stage. We previously demonstrated that optimal response to neoadjuvant chemotherapy was significantly associated with a decrease in the density of FoxP3^+^ regulatory T cells in the stroma of human HGSOC tumours.^38^ As we did not have access to treatment information for patients included in this cohort, we cannot rule it out as a potential confounding factor.

Importantly, we show that combining our six correlated types of immune infiltrates using PCA allowed us to transform inter-patient variations of the tumour immune landscape into independent, biologically interpretable PCs. We find that the main source of variation between patients is reflective of the quantity of concerted immune response. The second PC demonstrates that there is a pattern of significant variations in the CD8^+^ infiltrate of some patients that is independent and acting in opposition to the concerted immune response. The third PC measures the extent of negatively correlated epithelial and stromal infiltrates and is weighted differently by infiltrates. This supports our observation that immune exclusion is common, variable by infiltrate and shows that this is an additional effect to that of the concerted immune response.

The main limitation of our analysis of the ‘immunospace’ and its PCs is that we could only apply it to images that contain both epithelium and stroma. This reduced the size of the cohort we could analyse. With foresight, sampling could be designed to include both regions in TMAs and sectioning and imputation could be carried out for missing data. It is worth noting that being able to combine multiple correlated infiltrates and elucidate patterns and sources of variation will become even more important as many more (30+) immune markers are combined on single sections using imaging methods such as Hyperion. This method is particularly useful in that it also allows us to measure the strength of immune patterns occurring across multiple infiltrates. Analysing PCs also avoids some statistical issues that are associated with frequent co-correlation between different immune populations. Including correlated variables in a typical multivariable Cox regression survival model not only reduces interpretability but can also cause model results to be variable under bootstrapping as seen in the refined model of our cohort.

We found that the first PC was independently associated with survival. This result is consistent with a previous study showing that the simultaneous infiltration of tumours by different subsets of leucocytes (e.g. T cells, plasma cells, B cells), likely reflecting the establishment of a concerted immune response, gives a survival advantage to ovarian cancer patients.^5^ We used the PCs to model survival and found that a model including PC1 and PC5 was as predictive as individually modelled stromal CD45RO and CD68 infiltrates. Therefore, CD68 or CD45RO markers analysed in epithelium-adjacent stroma constitute the best single prognostic markers in our cohort.

In human lung adenocarcinoma, Mansuet-Lupo et al.^39^ showed that oncogenic mutations in the *TP53* gene were positively correlated with CD8^+^ infiltrate. In their study, intra-epithelial CD8^+^ T cell numbers and *TP53* status were both linked to prognosis with patients harbouring tumours with low CD8^+^ infiltrate and non-disruptive *TP53* mutation (associated with GOF) being linked to poorer survival. In glioblastoma, *TP53* mutations were also associated with increased immune infiltrate.^40^ In the present work, we integrated histological and genomic features to evaluate whether genetic alterations could be linked to different quantities of immune infiltration. We found no correlation between the nature of *TP53* mutations and the amount of CD8^+^ T cells, CD45RO^+^ memory lymphocytes and CD68^+^ macrophages in the stromal and malignant areas of 197 HGSOC tumours or the values of the PCs we derived. Overall, the exact nature of the relationship between subtypes of *TP53* mutations and immune infiltrate is still unclear and likely to vary across different cancers, as evidenced by two studies showing *TP53* GOF mutations to be associated with pro-tumour effects related to inflammation in glioblastoma^40^ and immunosuppression in lung adenocarcinoma.^41^

PTEN, another essential tumour suppressor, regulates the production of immunosuppressive cytokines by melanoma cells.^42^ In human melanoma, *PTEN* deletion was correlated with a decrease in infiltrating CD8^+^ T cells.^18^ In HGSOC, however, we observed no correlation between the expression level of PTEN and the number and/or localisation of CD8^+^, CD45RO^+^and CD68^+^ leucocytes in the tumour microenvironment or the PCs we derived. The discrepancy between the results obtained with HGSOC and melanoma tumours might lie in the fact that in our study loss of PTEN expression was only partial with tumours divided into expressing high or low levels of PTEN.

In contradiction to a previous work,^43^ we did not observe a significant correlation between the number of intra-epithelial CD8^+^ T cells and mutations in the *BRCA1* gene. As there were only 18 patients with a *BRCA1* mutation in this cohort, this is most likely related to a broad confidence interval as compared with the null population. We did however find a significant difference in intra-epithelial CD8^+^ T cell infiltration between patients with *BRCA1* and *BRCA2* mutations, with *BRCA1* patients having significantly higher infiltration than *BRCA2* cases. The discrepancy could also be linked to the automated and continuous quantitation method used in our study to quantify the immune infiltrate of tumours, as scoring in the previous study was done manually and in a stratified manner.^43^ Furthermore, we did not assess the methylation in the gene coding region for *BRCA1* and restricted our analysis to the identification of germline mutations in the *BRCA1* and *BRCA2* genes.

Our work shows that averaging immune infiltration over the whole tissue core could be as useful as current methods and potentially provides more prognostic information. Methods that average counts of immune cells across tissues are also simpler to implement than epithelial:stromal segmentation methods. Nonetheless, careful sampling of the tumour microenvironment with inclusion of both epithelium and stroma remains very important as both areas have independent prognostic significance.

Our results showing strong positive prognostic significance of stromal CD68^+^ infiltrate in HGSOC tumours warrants further investigation into the role and properties of CD68^+^ macrophages in HGSOC and also may caution against the use of new potential macrophage-depleting therapies.

## Supplementary information

Supplementary Figures and Tables

## Data Availability

The R markdown document containing the entire data set and allowing for reproducing all analyses performed in this manuscript is available online (https://bitbucket.org/jamesdbrenton/search-montfort/src/master/).
