# Supplementary material for: Combining measures of immune infiltration shows additive effect on survival prediction in high-grade serous ovarian carcinoma
Source: Br J Cancer. 2020 Apr 6;122(12):1803–10. doi: 10.1038/s41416-020-0822-x (PMC7283353; doi:10.1038/s41416-020-0822-x)
Supplement: Supplementary file 1 — Supplementary Figures and Tables [file 41416_2020_822_MOESM1_ESM.docx]

**Supplementary informations:**

This document contains supplementary figures and legends as well as supplementary tables.

**Supplementary Figures and legends:**

####
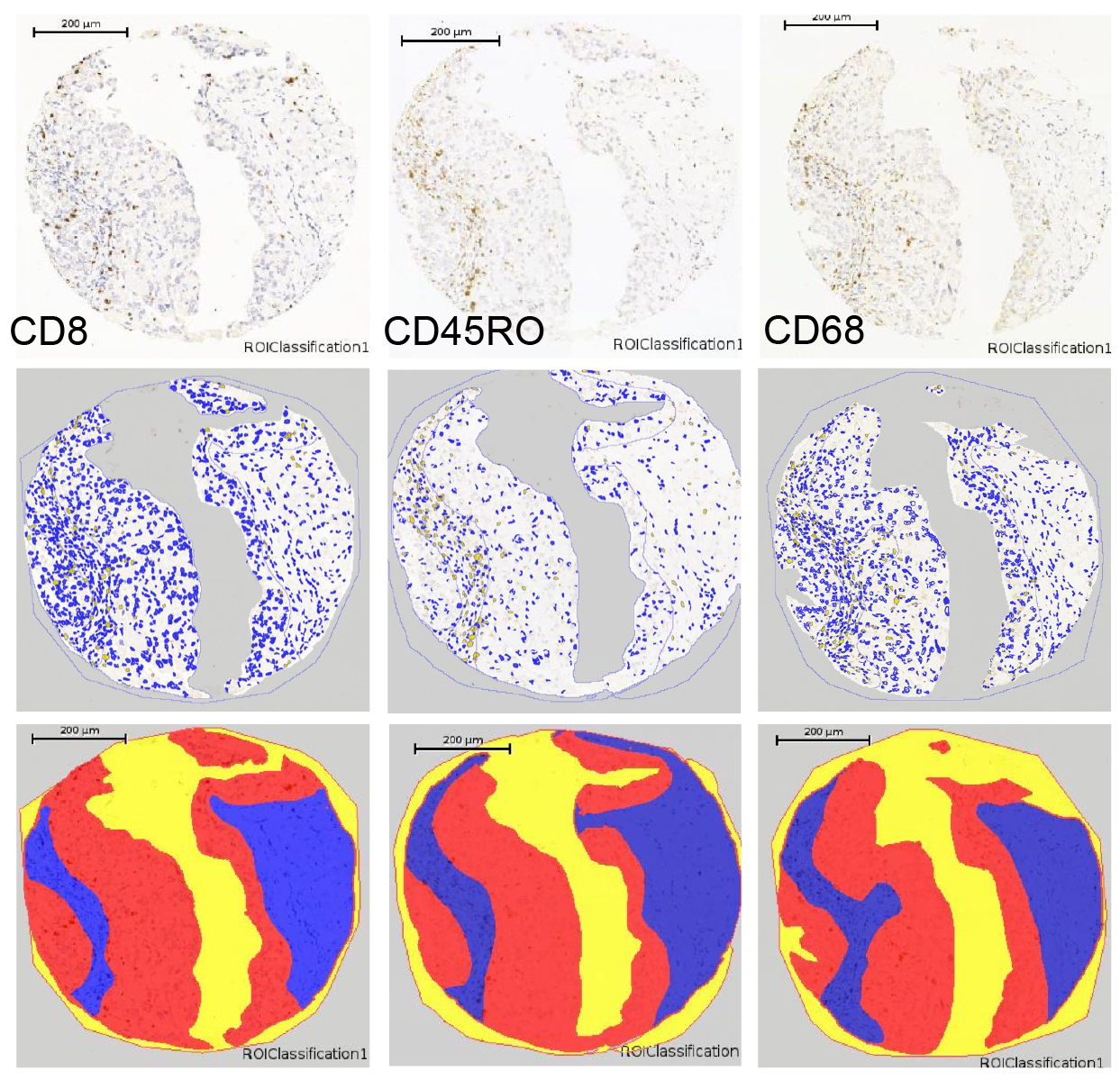


### Supplementary Fig. 1. Digital pathology analysis. Representative 0.6mm tissue microarray cores showing results of automated digital pathology analysis. Upper row shows haematoxylin and DAB stained sections. Middle row shows localisation of nuclei (blue) and cells of interest (brown staining, yellow labelling) following image analysis. Lower panel shows verified regions of epithelium (red) and stroma (blue). Yellow regions are undefined and not included in the analysis. Scale bars show 200 µm.

**
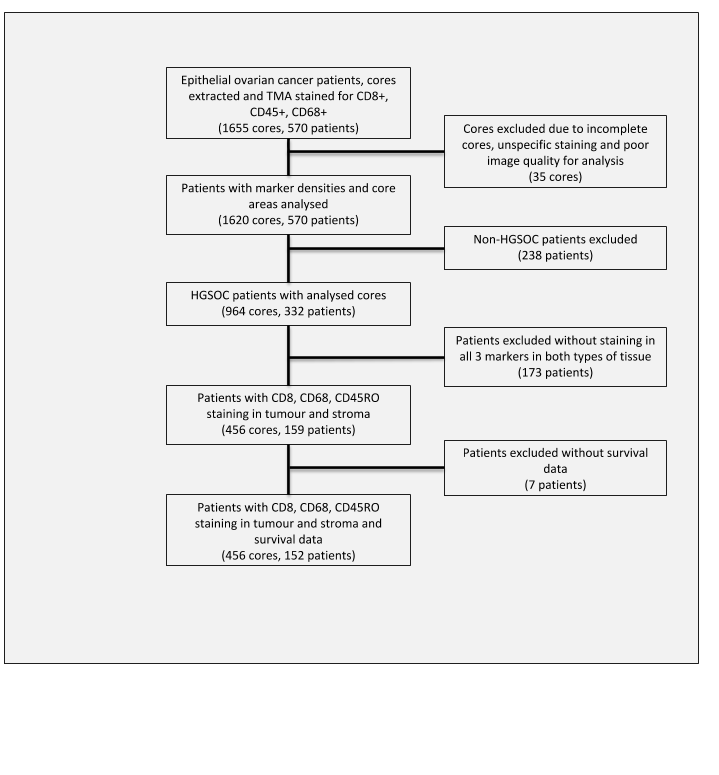
**

### Supplementary Fig. 2. REMARK diagram of patients analysed in principal component analysis and survival model comparisons.


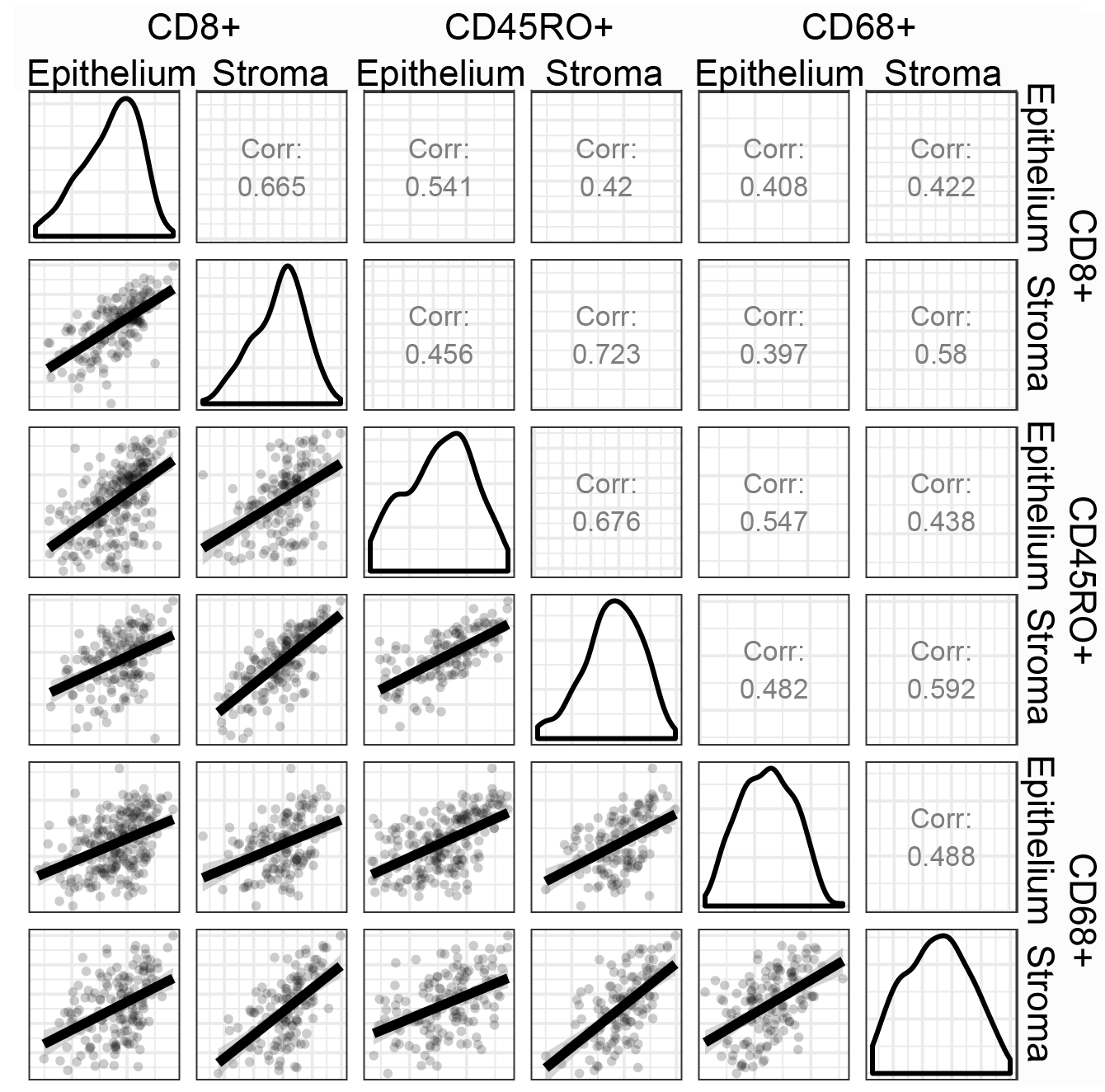


### Supplementary Fig. 3. Correlation between the CD8, CD45RO and CD68 infiltrates in HGSOC tumours. Pairwise correlation plots for infiltrate densities (CD8, CD45RO and CD68) within epithelial and stromal regions. Coefficients for the Pearson’s correlation using pairwise complete samples are shown. (See also Supplementary tables 4 and 5.) Count distributions are shown on the diagonal. Comparisons of the same infiltrate in epithelium and stroma are compared along the diagonal.


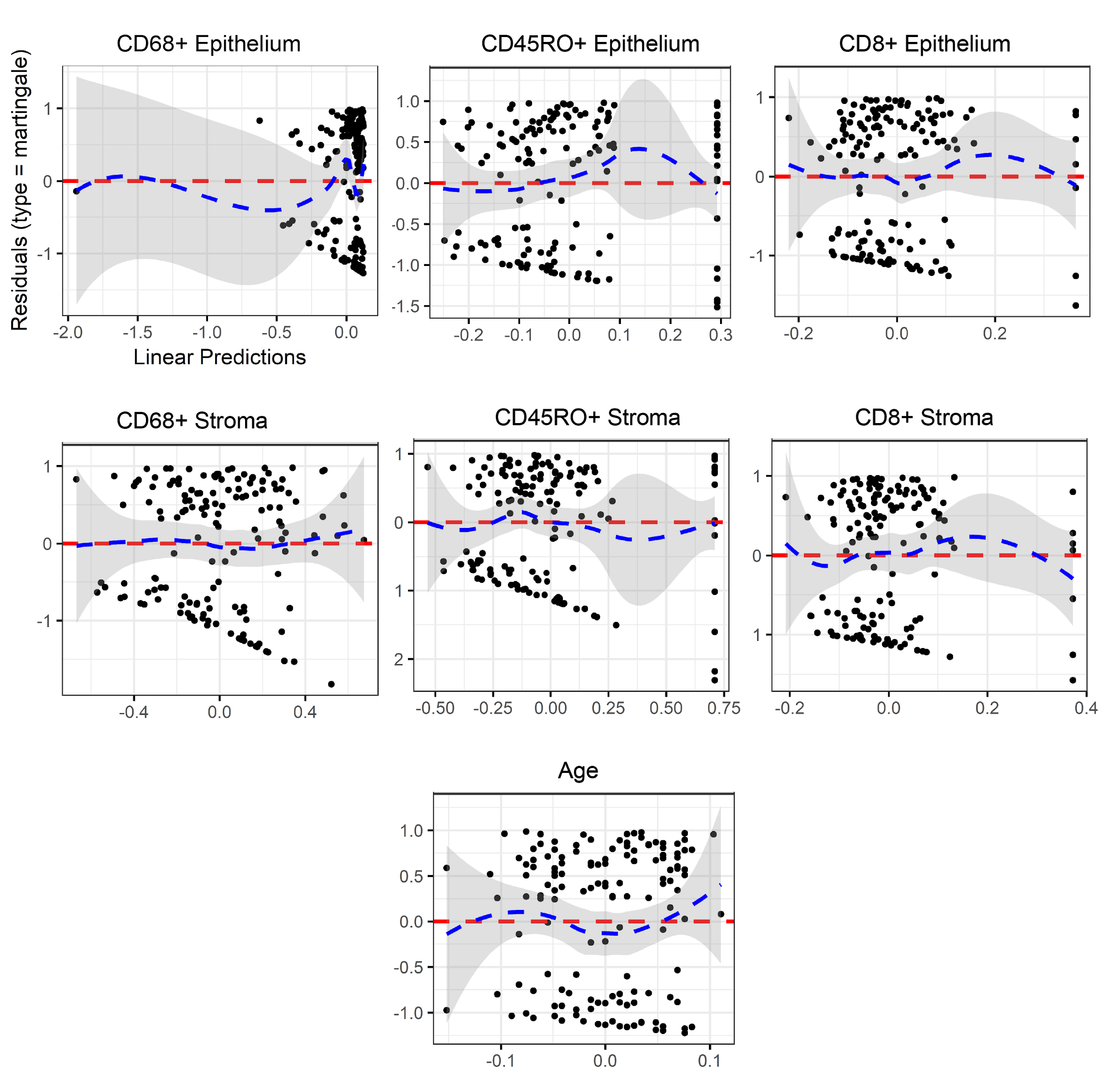


### Supplementary Fig. 4. Martingale residuals for each continuous predictor. Linear fit plotted in red.


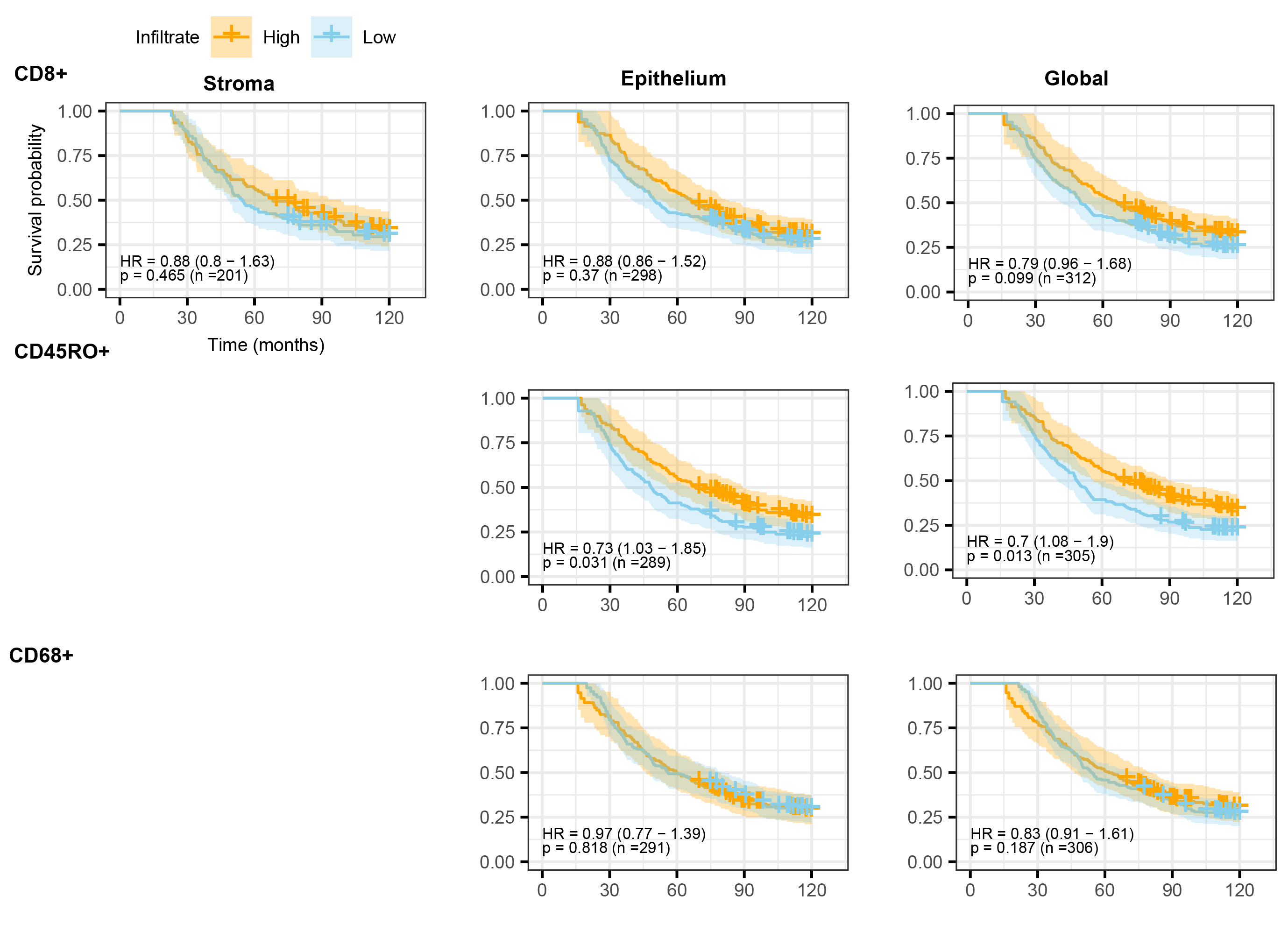


### Supplementary Fig. 5. Survival analysis of HGSOC patients relative to the density of CD8^+^ macrophages and CD45RO^+^ lymphocytes in their tumour biopsies. Kaplan–Meier survival curves split into two groups at the median density of CD8^+^ cytotoxic T cells and CD45RO^+^ infiltrates in stromal and epithelial areas as well as averaged across all tissues in the core. Survival curves were left truncated and right censored. Median entry to the study for all patients after diagnosis was 26.4 months. Median follow up time from diagnosis to exit or death was 105.1 months.


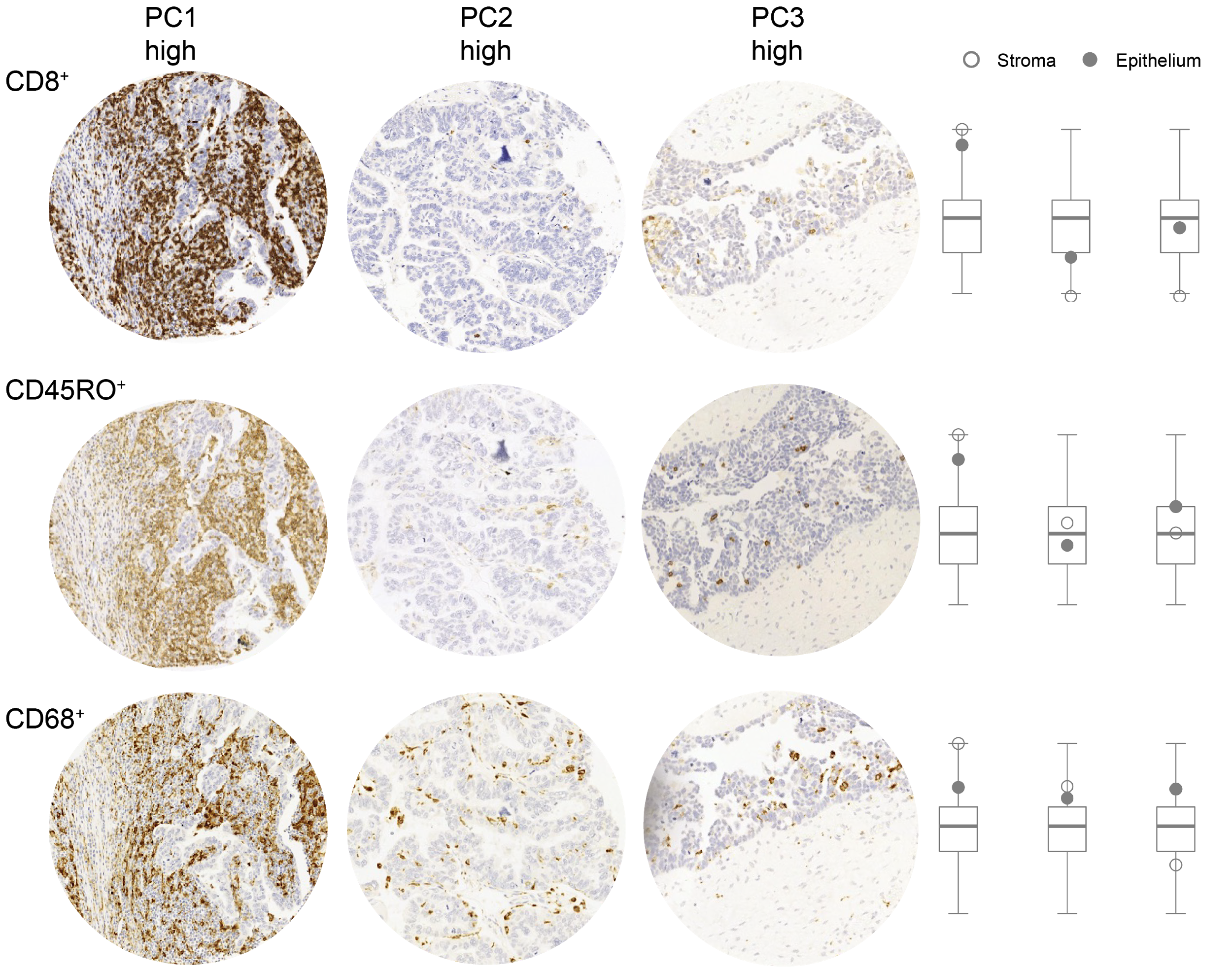


### Supplementary Fig. 6. Patient cores stained for CD8, CD45RO and CD68. Images are from patients with the highest PC1, PC2 and PC3 values. Images demonstrate the type of variance in immune infiltrate that the principal components explain. Boxplots show the distribution of densities for CD8, CD45RO and CD68 and the epithelial and stroma densities for each image are marked to show where cores lie in the distribution.


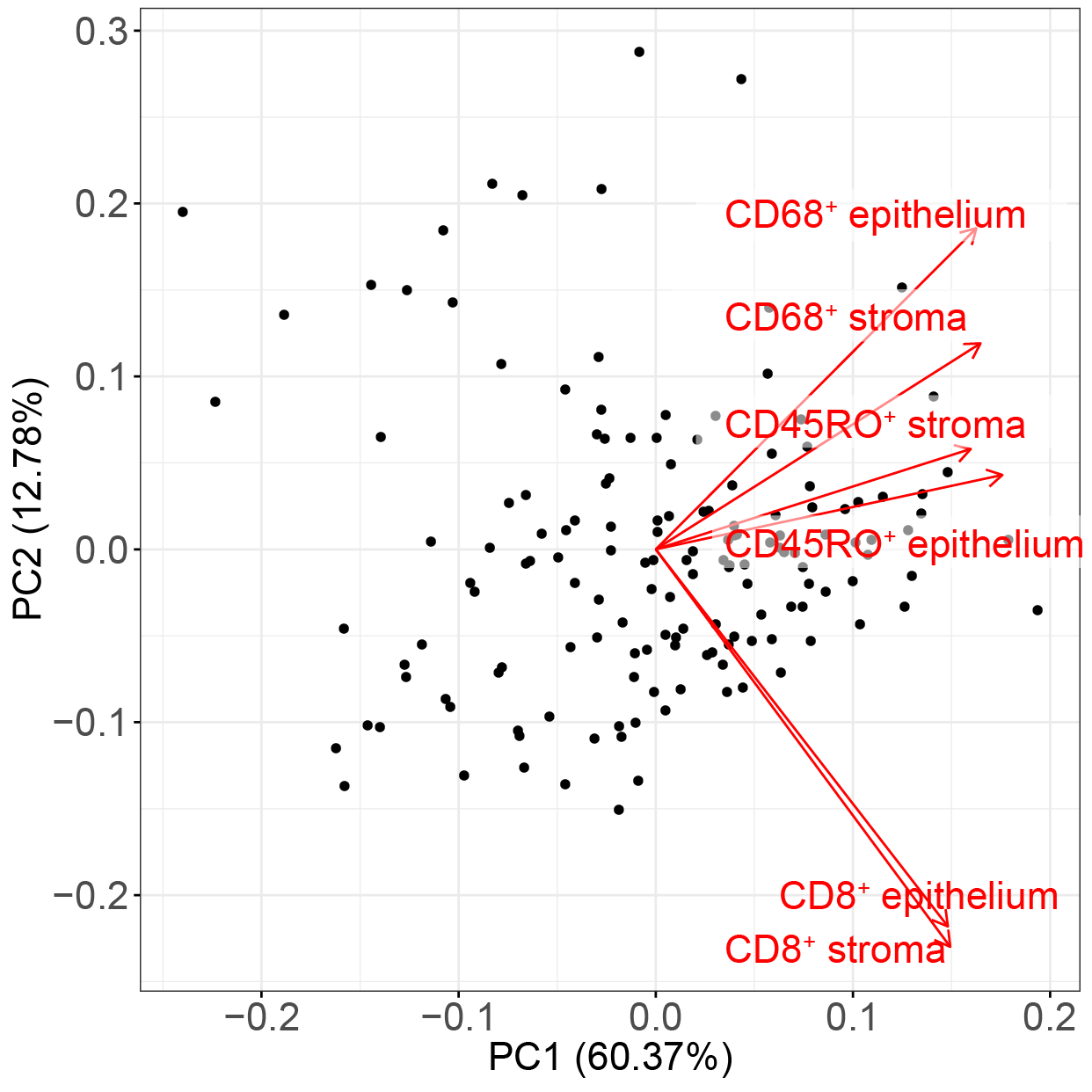


### Supplementary Fig. 7. Scatter plot of tumours against principal components 1 and 2 (PC1 and PC2) using normalized coordinates. Patients have values for each principal component based on a weighted sum (Suppl. Table 5). Red arrows indicate the weightings of the immune infiltrates in PC1 and PC2. The proportion of the variance attributed to each principal component is shown in brackets as a percentage.

###
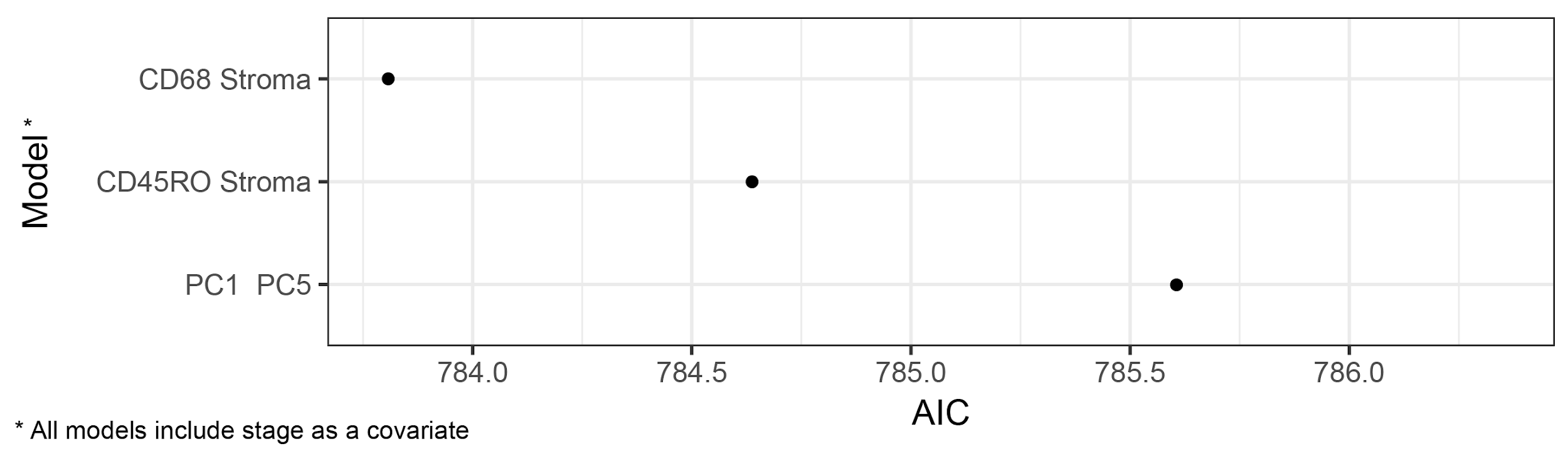


### Supplementary Fig. 8. Cleveland dotplot for the Akaike Information Criterion calculated for the best models of survival. Graph shows all models with a ΔAIC of <2. Optimum models have lower values. The accuracy of the fit of these three models is statistically indistinguishable when compared by their AIC.

**Supplementary tables:**

### Supplementary Table 1. HGSOC cancer patient characteristics

|  |  |  |
| --- | --- | --- |
| N |  | 332 |
| Median Age (IQR) |  | 58.0 (51.0-64.0) |
| Stage |  |  |
|  | localized | 64 (19.3%) |
|  | regional | 42 (12.7%) |
|  | distant | 202 (60.8%) |
|  | unstaged | 24 (7.2%) |
| *TP53* mutation |  |  |
|  | gof | 137 (55.2%) |
|  | lof | 94 (37.9%) |
|  | wild type | 17 (6.9%) |
|  | Not assessed | 84 |
| PTEN IF |  |  |
|  | High | 28 (18.1%) |
|  | Low | 127 (81.9%) |
|  | Not available | 177 |
| BRCA status |  |  |
|  | wild type | 256 (86.2%) |
|  | *BRCA1* | 18 (6.1%) |
|  | *BRCA2* | 23 (7.7%) |
|  | Not available | 35 |

###

### Supplementary Table 2. P values for the pairwise Pearson correlations of epithelial and stromal CD8^+^, CD45RO^+^ and CD68^+^ infiltrate in HGSOC tumours.

|  |  | CD8^+^ | | CD45RO^+^ | | CD68^+^ | |
| --- | --- | --- | --- | --- | --- | --- | --- |
|  |  | Epithelium | Stroma | Epithelium | Stroma | Epithelium | Stroma |
| CD8^+^ | Epithelium | 0 | 0 | 0.00E+00 | 1E-05 | 0.001 | 0.005 |
|  | Stroma | 0 | 0 | 1.20E-06 | 0 | 0.12 | 4E-05 |
| CD45RO^+^ | Epithelium | 0 | 1E-07 | 0 | 0 | 5E-07 | 0.0003 |
|  | Stroma | 2E-06 | 0 | 0 | 0 | 0.01 | 2E-06 |
| CD68^+^ | Epithelium | 0.0003 | 0.12 | 0 | 0.004 | 0 | 0.04 |
|  | Stroma | 0.0013 | 6.1E-06 | 5.6E-05 | 2E-07 | 0.02 | 0 |

###

### Supplementary Table 3. P-values for the association of each variable to survival using univariable Cox regression analyses demonstrating functional form is approximately log linear.

|  | Linear | Cubic splines | Log(base 10) |
| --- | --- | --- | --- |
|  | p-value | p-value | p-value |
| Age at diagnosis | 0.18 | 0.36 | 0.24 |
| CD8+ epithelium | 0.43 | 0.36 | 0.25 |
| CD8+ stroma | 0.40 | 0.17 | 0.64 |
| CD68+ epithelium | 0.44 | 0.57 | 0.63 |
| CD68+ stroma | 0.09 | 0.06 | 0.009 |
| CD45RO+ epithelium | 0.30 | 0.09 | 0.07 |
| CD45RO+ stroma | 0.07 | 0.016 | 0.002 |

###

### Supplementary Table 4. Immune infiltrate and survival. Cox proportional hazard regression for cores with malignant epithelial tissue only. Multivariable analysis includes stage.

|  |  | Univariable | | Multivariable* | |
| --- | --- | --- | --- | --- | --- |
|  | Cases | HR | p-value | HR | p-value |
| CD8^+^ | 111 | 0.84 | 0.27 | 0.70 | **0.047** |
| CD45RO^+^ | 110 | 0.98 | 0.89 | 0.96 | 0.78 |
| CD68^+^ | 80 | 1.21 | 0.47 | 1.27 | 0.39 |

###

### Supplementary Table 5. Composition of the principal components across all infiltrates. Figures in brackets indicate proportion of total variance.

|  | PC1  (60%) | PC2  (13%) | PC3  (9%) | PC4  (8%) | PC5  (5.5%) | PC6  (4.5%) |
| --- | --- | --- | --- | --- | --- | --- |
| CD8^+^ epithelial density | 0.38 | -0.56 | 0.58 | -0.10 | 0.40 | -0.21 |
| CD8^+^ stromal density | 0.38 | -0.59 | -0.47 | 0.27 | -0.21 | 0.43 |
| CD68^+^ epithelial density | 0.41 | 0.47 | 0.37 | 0.11 | 0.09 | 0.67 |
| CD68^+^ stromal density | 0.42 | 0.30 | -0.29 | 0.56 | 0.36 | -0.45 |
| CD45RO^+^ epithelial density | 0.45 | 0.11 | 0.21 | -0.07 | -0.78 | -0.36 |
| CD45RO^+^ stromal density | 0.41 | 0.15 | -0.42 | -0.77 | 0.22 | -0.03 |

### Supplementary Table 6. Immune principal components and survival. Cox proportional hazard regression for principal components as predictors. Multivariable analysis includes stage.

|  | Univariable | | Multivariable | |
| --- | --- | --- | --- | --- |
|  | HR | p-value | HR | p-value |
| PC1 | 0.89 | 0.024 | 0.88 | 0.016 |
| PC2 | 0.94 | 0.61 | 0.92 | 0.52 |
| PC3 | 1.20 | 0.20 | 1.23 | 0.18 |
| PC4 | 1.14 | 0.43 | 1.07 | 0.69 |
| PC5 | 0.82 | 0.31 | 0.74 | 0.11 |
| PC6 | 1.25 | 0.23 | 1.22 | 0.33 |
